# Beyond borders: Decoding the influence of economic development, money confidence, financial market, and purchasing power on currency internationalization

**DOI:** 10.1371/journal.pone.0317765

**Published:** 2025-02-11

**Authors:** Zibin Cao, Weini Soh, Nazrul Hisyam Ab Razak, Bany Ariffin Amin Noordin

**Affiliations:** 1 School of Economics and Management, Zhoukou Normal University, Zhoukou City, Henan Province, China; 2 School of Business and Economics, Universiti Putra Malaysia, UPM Serdang, Seri Kembangan, Selangor Darul Ehsan, Malaysia; Ala-Too International University, KYRGYZSTAN

## Abstract

Due to dedollarization and deglobalization trends, countries are pursuing currency diversification to reduce reliance on the U.S. dollar and mitigate currency risks. The research on the drivers of currency internationalization still faces problems such as small sample sizes, fewer methods, and incomplete theoretical frameworks. This study aims to investigate the effects of economic development, money confidence, and the financial market on currency internationalization. It also explores whether purchasing power mediates the relationships between the first two exogenous variables and currency internationalization. The Partial least squares structural equation modeling (PLS-SEM) method is used to analyze secondary data from 9 of the 10 most used currencies (excluding the euro) from 2000 to 2020 to examine these relationships. The findings show that economic development and money confidence have negative and significant relationships with currency internationalization, while financial market and purchasing power have positive and significant relationships with currency internationalization. The relationships between economic development and currency internationalization, as well as between money confidence and currency internationalization, are both mediated by purchasing power. These mediation effects are partially complementary mediation effects. Accordingly, to promote currency internationalization, this study recommends governments should adopt policies to develop the financial market, increase openness, and reduce capital controls. It also highlights the importance of managing inflation, diversifying reserve assets, and maintaining a flexible exchange rate to prevent currency depreciation. This study is limited by the exclusion of the euro, reliance on hard data, a small sample size, and a narrow focus on economic factors.

## 1. Introduction

The concept of currency internationalization has attracted great attention in past decades reflecting the global shift from nationalism to globalism and the expansion of international trade [1]. Currency internationalization, also known as an international currency, refers to a currency normally used and accepted in international trade, transactions, settlements, and investments outside its issuing country [2]. This phenomenon is intrinsically linked to the core functions of currency. While a domestic currency primarily serves as a unit of account, a medium of exchange, and a store of value within a specific country, an international currency extends these roles across global markets [3]. Issuing an international currency can bring substantial benefits to the issuers, including prestige, leverage, and seigniorage [1]. Meanwhile, it brings convenience for tourists and financial institutions by reducing transaction costs and improving trade efficiency [4]. For developed economies, currency internationalization is not merely a byproduct of economic strength but a strategic tool for enhancing national influence and reputation. Therefore, these countries need to adopt targeted political and economic policies to expand the use range of their currencies and maintain their economic competitiveness on the international stage.

The growing tendency of dedollarization and deglobalization have significantly changed the dynamics of international currencies by challenging the dominance of the U.S. dollar and reshaping global economic orders. Because of economic instability and uncertainties led by Brexit, the COVID-19 pandemic, and the Russia-Ukrainian War, countries tend to implement conservative economic policies and prefer to use various currencies in cross-border trade and transactions to decrease foreign exchange risk and reduce their reliance on the U.S. dollar [5]. According to the statistics released by the International Monetary Fund (IMF), the share of the U.S. dollar in global foreign exchange reserves has significantly declined from 65.46% in the first quarter of 2016 to 59.39% in the third quarter of 2024. This trend has coincided with an increasing preference for gold as a safe-haven asset among central banks, potentially driven by a strategic reduction in reliance on the U.S. dollar [6]. Since 2022, countries such as China, Turkey, and India have notably expanded their gold reserves, reflecting their preference for risk avoidance. Central banks in developed countries, including Singapore, the United Kingdom, Poland, and Qatar, have increased the proportion of gold reserves within their foreign exchange reserves.

Concurrently, promoting national currency usage and regional monetary cooperation are considered good alternatives to the U.S. dollar. Emerging economies, such as the BRICS countries, are actively promoting the use of their local currencies in bilateral trade to reduce reliance on the U.S. dollar. Similarly, European countries show a preference for the euro in extra-EU goods trade. According to data from the European Commission, in 2023, 46% of extra-EU trade in goods was conducted in euro, compared to 42% in the U.S. dollar. In export transactions, the euro constituted approximately 52% of the total, significantly exceeding the 32% share occupied by the U.S. dollar. Additionally, several Asian economies in ASEAN, such as Indonesia, Malaysia, Thailand, Philippines, and Singapore, are taking actions to increase regional currency cooperation and promote local currency use in trade and investment by establishing Local Currency Settlement (LCS) framework and Local Currency Transaction (LCT) system. The diversification of international currency in global trade is being driven by technological advancements, including the rise of digital currencies and central bank digital currencies (CBDCs), and is driving the diversification of international currency in global trade. More than 130 countries’ central banks have investigated digital currencies to accelerate dedollarization and safeguard national interests; 66 of them are in the advanced phase of exploration based on Atlantic Council data.

Under this background, the previous international financial system is gradually collapsing, and it becomes difficult to maintain a dominant currency, so building a multi-currency system to replace the existing single-currency system has aroused great interest among scholars [7,8]. Thus, understanding the key drivers of currency internationalization can help countries enhance international use of national currencies and reduce exposure to currency risk.

Although many papers have analyzed the key drivers of international currency, few have achieved a consensus. A country’s currency status in the world is influenced by economic, political, cultural, and military factors, with macroeconomic performance undoubtedly exerting the greatest impact among them [4]. A conceptual framework for currency internationalization from the economic field was proposed by Chinn and Frankel [9], including four determinants, which are output and trade, money confidence, financial market, and network externalities. Except for network externality, measuring inertia, the other three factors are all important components of macroeconomic performance. Chinn and Frankel [9] have conducted research on the pound, the dollar, and the euro and anticipated the euro and RMB will become the US dollar’s big competitors in the future. However, the concept of output and trade primarily reflects a country’s economic size while overlooking its economic growth rate, both of which are essential to the currency internationalization process. This is exemplified by the case of the Chinese RMB, whose internationalization process is closely linked to China’s economic status as the world’s second-largest economy and its impressive annual economic growth rate of about 6-7%. Therefore, economic development, which includes both economic size and economic growth rate, can be regarded as a key factor driving currency internationalization instead of output and trade measure.

Purchasing power refers to the ability of a currency to buy goods and services, or the amount of goods and services that one unit of currency can buy [10]. The inner value of money refers to the purchasing power in a certain country and the outer value of money refers to the exchange value of a country’s currency to other currencies, always known as the exchange rate. Inflation rate and purchasing power are inversely correlated. Getting the purchasing power of foreign currency is the main motivation for holding it, as it allows people to afford goods and services. The internal value of money, determined by the currency itself, can affect its external value, which is its purchasing power relative to other goods [11]. Factors such as economic development [12], currency stability [13], future expectations [14], and currency demand [15] influence the external value of money. An increase in purchasing power, indicating a rise in value, leads to currency appreciation and currency internationalization ultimately. Therefore, purchasing power might be a potential channel via which currency internationalization is influenced by economic development and money confidence.

Undoubtedly, the U.S. dollar has still maintained a large market share in international trade and payment at about 46.64% based on SWIFT data in January 2024. Since China started the first cross-border RMB trade settlement service in 2009, China has made great progress in RMB internationalization. RMB was added to the SDR basket on October 1st, 2016, as the fifth currency and it became the 4^th^ largest currency in the world with a market share of 4.51% and even ranked the 3^rd^ in trade finance market in January 2024. Inspired by Chinese policies, India and Russia also strive to enhance the international status of the Rupee and Ruble [16]. Therefore, a looming question arises regarding the relationship between economic performance and currency internationalization, as well as its implications for emerging economies seeking to promote currency internationalization. Exploring the key drivers of currency internationalization, along with the mediation effects between these factors and international currency, provides valuable insight for evaluating which currencies have the potential to gain world recognition. By examining the three key aspects of economic performance, countries can understand the conditions necessary for a currency to obtain global acceptance and identify the barriers that might hinder its global usage. This will help to predict not only which currencies are qualified for internationalization but also the challenges they must overcome to achieve this target.

Previous papers usually investigate currency internationalization by econometric models, especially multiple regression models, and limited studies have evaluated this issue by using the partial least squares structural equation modeling (PLS-SEM) method, particularly in terms of evaluating potential mediation effects between these drivers and currency internationalization. With only 21 years of data from 9 countries in this paper, the sample size is indeed relatively small for multiple regression analysis. However, the PLS-SEM method offers several advantages, including fewer restrictions regarding data distribution and small sample size, and it can still provide reliable results even with a small sample [17]. Moreover, the PLS-SEM is particularly effective in handling latent variables and indicators, allowing researchers to capture the multidimensionality of currency internationalization drivers accurately. This approach also examines the mediation effects of purchasing power on the relationships between economic development, money confidence, and currency internationalization and analyzes the complicated pathways through which various key drivers influence currency internationalization [18]. Finally, it is useful for prediction accuracy and relevance, offering a comprehensive framework to elucidate the driving forces and mediating mechanisms underlying currency internationalization [19].

This study builds on previous research by expanding currency selection to include a broader range of currencies. Early studies normally focused on reserve currencies in the SDR basket, resulting in limited research samples, and ignored other promising currencies at the top of the rankings. For this study, due to the unavailability of euro data, 9 of the 10 most used currencies in the global financial market were selected, including the Australian dollar, Canadian Dollar, Chinese RMB, U.S. Dollar, U.K Pound Sterling, Japanese Yen, Swiss Franc, Sweden Krona, and Hong Kong Dollar. To address these research gaps, three research questions are determined as follows: **RQ 1:** What are the key drivers of currency internationalization? **RQ 2:** Do mediation effects of purchasing power exist on the relationships between economic development and currency internationalization and money confidence and currency internationalization? **RQ 3:** If mediation effects exist, what kind of mediation effect are they?

To solve these problems, based on currency substitution theory and purchasing power parity theory, a theoretical framework integrates three exogenous variables economic development, money confidence, financial market, a mediator variable purchasing power, and an endogenous variable currency internationalization. This study employs the PLS-SEM method, including formative measurement model assessment, structural model assessment, and mediation effect assessment, to analyze the dynamic relationships between key drivers and currency internationalization. Specifically, it explores the potential mediation effects between economic development and currency internationalization, as well as between money confidence and currency internationalization.

This paper is arranged as follows. Chapter 2 outlines the theoretical background and proposes the hypotheses development. Chapter 3 explains the research methodology, which includes research design, data collection, sampling, data analysis, and methodology justification. Chapter 4 presents the main findings of this study, including descriptive statistics, formative measurement model assessment, structural model assessment, and mediation effect analysis. Chapter 5 introduces the discussion of the study. Chapter 6 contains the conclusion, implications, policy suggestions, limitations, and suggestions for future studies.

## 2. Theoretical background and research hypotheses

### 2.1 Currency substitution theory

The currency substitution (CS) theory was established by Chetty [20] and expanded by Ramirez-Rojas [21] and Calvo and Gramont [22]. Currency substitution means when a country’s citizens lose trust in their domestic currency or if the return on that currency is insufficient, they can substitute it wholly or partially with a foreign currency satisfying the functions [23]. Developing countries rely more on international currencies, especially the US dollar, which shows an asymmetrical currency substitution situation, while developed countries are the opposite, showing similar currency substitution. There are two types of currency substitution: indirect substitution and direct substitution. If investors desire liquidity and hope to change their currencies into other currencies, it is called indirect currency substitution. If there is a competitive dynamic between different currencies regarding their role as means of payment, it can be regarded as direct substitution [24].

### 2.2 Purchasing power parity theory

The purchasing power parity (PPP) theory was proposed by Cassel [25]. According to purchasing power parity, the same goods and services should cost the same in different countries without transaction costs and other considerations. To precisely determine the relative purchasing power of different currencies, the purchasing power parity is used to compare their values. This concept of purchasing power parity is widely used to compare the economic development of various countries [26], solve global poverty problems [27], and analyze foreign exchange rates [28]. Purchasing power, often linked to currency appreciation, reflects a currency’s increased ability to acquire more domestic goods and services as well as its stronger position in the foreign exchange market. Conversely, currency depreciation reduces purchasing power, making domestic goods more expensive and weakening the currency’s value in international markets, which limits the ability to purchase foreign goods and services [29].

### 2.3 Currency internationalization

International currency refers to a currency utilized beyond its issuing country for trade, investment, and reservation. It meets all the monetary functions of domestic currency and can be considered a special domestic currency. In academic research examining the factors that contribute to a currency’s widespread acceptance as an international currency and the potential emergence of new international currencies, the degree of currency internationalization is commonly analyzed as a dependent variable. Based on CS theory, Chinn and Frankel constructed a research framework for currency internationalization [9], including output and trade, money confidence, financial market, and network externalities. More international trade and macroeconomic stability contributed to the improved international status of currency [2]. Some studies have used the euro as an example, arguing that economic integration and greater economic ties will lead to using the same currency [30]. Liu et al. [31] examined the Chinese RMB internationalization process and identified economic size, trade networks, and financial market development as significant factors influencing the RMB’s international use. Cui et al. [32] found economic size, money confidence, financial market, and network externalities can influence currency internationalization and international competitiveness can be regarded as another influencing factor. The relationship between local currency debt and currency internationalization dynamics were examined, highlighting the importance of financial market development and technological innovation in currency internationalization [33].

### 2.4 Economic development

Economic development is always defined as the sustained improvement in a region’s or typically a nation’s general living conditions and economic well-being [34]. It plays a critical role in sharing international currency dynamics, influencing exchange rates, trade balances, and global financial stability. The countries with international currencies are always the countries with great power, suggesting that currency internationalization is associated with economic development [35]. Positive trade balances, increased investor confidence, and good economic policies all contribute to a country’s currency strength as its economy expands. A nation’s development level is strongly correlated with currency performance, showing a close relationship between the macroeconomic situation and the exchange rate mechanism [36]. Economic development is a key driver of currency internationalization. Previous studies found that there was a positive relationship between economic size and international use of its currency [32]. This indicates that as economic size grows, the process of currency internationalization will increase. The euroization and RMB internationalization are inseparable from their larger economies and the higher economic growth of China has also boosted its currency usage. The slowdown in economic development will also decrease the process of currency internationalization process [37]. GDP, FDI, and GDP growth rate are commonly used measures to capture economic development. GDP and its growth contribute to short-term currency appreciation, reinforcing its utility in international markets [38]. FDI has a significant influence on offshore currency usage, as vehicle currency use grows by 3.54% with every 1% rise in bilateral FDI [31]. The country’s economic development plays an important part in currency internationalization. Hence, the hypothesis is proposed:

H1: There is a significant relationship between economic development and currency internationalization.

### 2.5 Money confidence

Money confidence is the second important driver of currency internationalization. Stable currencies are preferred by people and nations because they can mitigate financial risks and maintain value over time [37]. Money confidence is only generated when a currency is deemed to possess a stable value within a specific timeframe. Reducing currency volatility means controlling transaction costs [39]. Maintaining the fundamental stability of currency value at a fair and sustainable level is the central banks’ major objective, benefiting both the nation and the global economy together. Recent studies examine the relationship between money confidence and currency internationalization. By using an experimental method, Cardozo et al. [40] introduced how the emergence of international currencies relies on confidence in the issuing country’s monetary stability and low inflation rates. Furthermore, empirical findings reveal that confidence in currency stability directly impacts trade efficiency and global currency circulation. Gold reserve and foreign exchange reserve are two important components of money confidence, maintaining a country’s economic stability and enhancing the trustworthiness of its currency. Gold reserve is considered high-quality hedging assets due to its exceptional security features and constitutes a significant portion of national asset allocations [6]. Previous papers have investigated that U.S. dollar internationalization was strongly linked to its gold reserve while euro global usage had a cointegration relationship with gold reserves in European countries [41]. In recent years, emerging economies, such as China, Russia, and India, have increased their gold reserves to reduce their dependence on the U.S. dollar and mitigate the impact of potential sanctions, thereby promoting the international use of their currencies [6]. Foreign exchange reserve is also held by central banks to protect against economic fluctuations, maintain price and financial stability, and improve export competitiveness [42]. The supply and demand of a currency are influenced by changes in its foreign exchange reserves. Abundant foreign exchange reserves give governments a benchmark and a foundation for modifying monetary policies. The diversification of foreign exchange reserves enables the country to reduce the reliance on a dominant currency, like the U.S. dollar, and enhance the global usage of other alternative currencies [43]. Accordingly, the second hypothesis is proposed:

H2: There is a significant relationship between money confidence and currency internationalization.

### 2.6 Financial market

The third key driver to promote currency internationalization is an open, deep, and well-developed financial market [32]. A prosperous mature financial market enhances liquidity, reduces transaction costs, and increases currency exchange. A study on the internationalization of emerging market currencies illustrated by reducing “original sin”—the incapacity of nations to borrow internationally in their currency, financial market growth helps to promote currency internationalization [39]. Moreover, research on the Chinese RMB internationalization demonstrates that the RMB usage throughout the world has benefited from developments in China’s financial market [31]. Four indicators are commonly used to capture the development of the financial market, which are private credit to GDP, stock market capitalization to GDP, Chinn-Ito index, and M2 over GDP. A widely used indicator to evaluate the debt market is private credit to GDP. A regression model was utilized to analyze the influence of the credit market and exchange rate on currency internationalization, revealing that they did not significantly influence the process [44]. The percentage of private credit to GDP, measuring financial development, was negatively related to the invoicing share of an international currency [45]. However, it was also evaluated to have a statistically positive relationship with currency usage in trade [31]. Stock market capitalization to GDP is an indicator used to examine the valuation of a country’s stock market relative to its economic output, capturing the depth of the financial market. It was proven to have a significantly positive relationship with the reserve currency share and the use of international currency [46]. Chinn-Ito index (also known as KAOPEN) is an indicator to evaluate the openness of the financial market. An unrestricted financial market fosters currency internationalization, with a strong relationship between KAOPEN and currency internationalization [45]. M2 over GDP is an indicator for countries to evaluate the depth of the financial market. It showed a positive relationship with global currency use [47]. Accordingly, the third hypothesis is given:

H3: There is a significant relationship between the financial market and currency internationalization.

### 2.7 Purchasing power

The purchasing power of a currency is generally regarded as the amount of goods and services that can be purchased by a unit of currency [10]. It is seen as a measure of the value of money. For different currencies, purchasing power explains why various currencies differ in value and why some currencies are more welcomed than others to become international currencies. The goal of holding a foreign currency is to acquire the purchasing power of that money, which people can utilize to afford products and services. If the purchasing power of a currency increases, which means the appreciation of the currency, people will be more willing to hold this currency, which will lead to its currency internationalization [11]. The complex relationships between currency value fluctuations, external debt, and economic growth were found in currency devaluation dynamics. This study shows the significance of currency value fluctuations can influence a country’s economic performance and its currency’s international position [33]. The innovation-driven economic growth leads to increased purchasing power through the creation and redistribution of financial resources, enabling higher demand for goods and services [48]. The welfare benefit gained from being an international currency depends on the inflation rate, usually measured by Consumer Price Index (CPI), and the Producer Price Index (PPI). Residents prefer the predominant international currency to have the lowest inflation rate, suggesting it has more purchasing power [49]. The Big Mac Index (BMI), which compares the price of hamburgers across countries, is also widely used to compare the purchasing power among currencies [50]. Hence, the fourth hypothesis is given:

H4: There is a significant relationship between purchasing power and currency internationalization.

### 2.8 Mediation effect

The purchasing power of money includes the inner value of money and the outer value of money. The inner value of money refers to the purchasing power in a certain country and the outer value of money refers to the exchange value of a country’s currency concerning other currencies, always known as the exchange rate. The internal value of a currency is inversely related to the inflation rate, and changes in the inner value can influence its outer value. The inner value of money is determined by money itself while the outer value is determined by the purchasing power relative to all other items [10]. Many scholars proved that the outer value of money is influenced by many factors such as economic development [12], currency stability [13], future expectations [14], and currency demand [15]. Economic size and macroeconomic stabilities were verified to promote the status of a currency and lead to currency appreciation. Higher purchasing power contributes to the currency internationalization process. Furthermore, money confidence is often reflected by a country’s precious metal reserves and foreign exchange reserves. Analyzing the prices of precious metals and seven local currencies reveals negative correlations between these two. Precious metals should not be utilized to hedge against local currency risk since they are unreliable tools for maintaining a local currency’s external purchasing power [6]. Therefore, purchasing power serves as a meaningful pathway through which economic development and money confidence influence currency internationalization. Four related hypotheses are listed below:

H5: There is a significant relationship between economic development and purchasing power.

H6: There is a significant relationship between money confidence and purchasing power.

H7: There is a mediation effect of purchasing power on the relationship between economic development and currency internationalization.

H8: There is a mediation effect of purchasing power on the relationship between money confidence and currency internationalization.

## 3. Research methodology

### 3.1 Research design

**Fig 1:**
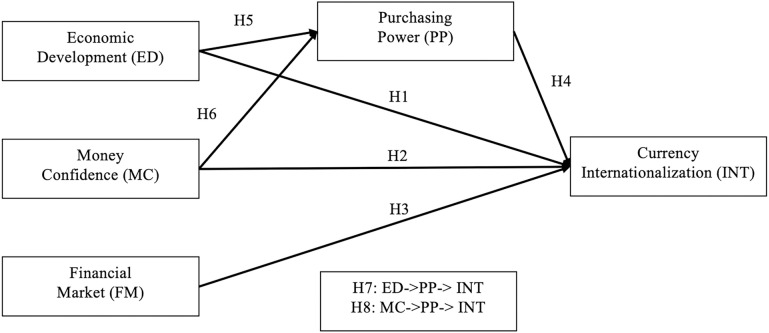
The research framework of this study.

This study employs a quantitative research approach and utilizes the PLS-SEM method to examine the direct and indirect relationships between key drivers and currency internationalization. The PLS-SEM method is chosen because it is effective in handling complex relationships, small sample sizes, and non-normally distributed data, which makes it suitable for analyzing the dynamics between macroeconomic performance and international currency. The research framework, as illustrated in Fig 1, evaluates three exogenous variables (economic development, money confidence, and financial market) as direct drivers of the currency internationalization process. Additionally, it identifies purchasing power as a potential mediator via which currency internationalization is influenced by economic development and money confidence. All the variables involved are abstract and difficult to capture, so using only one or two proxies to evaluate is far from enough. In this paper, each variable is well segmented, and more indicators are covered in each variable to improve the accuracy of the research.

### 3.2 Data collection

This study relies on yearly secondary data consisting of macroeconomic and financial indicators at the country level from 2000 to 2020. All the data are hard data, which are collected from reliable and accessible databases, including the CEIC database, the World Bank website, the Chinn-Ito website, and the KOF Swiss Economic Institute website. This study includes three exogenous variables, which are economic development (ED), money confidence (MC), and financial market (FM), a mediator variable purchasing power (PP), and an endogenous variable currency internationalization (INT). Each construct is measured by at least 2 indicators to reduce potential measurement error and promote reliability and predictive validity. The indicators are chosen because they shape and form the basic idea of the constructs in past papers. Data standardization was performed to eliminate the scale differences and ensure consistency across variables, normalizing the data to a range from 0 to 1. This allows for a more accurate comparison and analysis of the relationships between these constructs. The data span from 2000 to 2020, chosen for several reasons. First, some indicators were only published on an annual basis without corresponding monthly or quarterly data. Second, some indicators could only be collected from the year 2000 while some indicators for Hong Kong were not publicly available. Keeping the same time interval for all nine issuers made it easy to collect and compare data. Yearly data presents a holistic evaluation of a country’s economic performance over a fixed period, making it easier to analyze long-term trends and fluctuations. Given the significant transformations experienced by many countries in the past two decades, especially China, this time frame was deemed appropriate for the study. Table 1 presents the definitions and data sources of all constructs and indicators.

**Table 1:** The summary of constructs, indicators, definitions, and data sources.

Construct	Indicator	Definition	Source
Economic Development (ED)	GDP Share (GDPS)	The country’s GDP over the global GDP	World Bank
	GDP Growth Rate (GGR)	The country’s GDP growth rate	World Bank
	FDI (FDI)	The country’s foreign direct investment	World Bank
	Real GDP (GDP)	The inflation-adjusted value of a country’s GDP	World Bank
Money Confidence (MC)	Gold Reserve (GR)	The gold reserve held by the central bank	CEIC
	Foreign Exchange Reserve (FXR)	The foreign exchange reserve held by the central bank	CEIC
Financial Market (FM)	Private Credit to GDP (PC)	The domestic credit to the private sector by banks over the country’s GDP	CEIC
	Stock Market Capitalization/GDP (SMC)	The country’s stock market capitalization over the country’s GDP	World Bank and CEIC
	KAOPEN (KAOPEN)	The Chinn-Ito index	Chinn-Ito website
	M2/GDP (M2)	The country’s M2 over the country’s GDP	CEIC
Purchasing Power (PP)	Consumer Price Index (CPI)	The country’s consumer price index	CEIC
	Big Mac Index (BMI)	The country’s mac burger price	The Economist
	Producer Price Index (PPI)	The country’s producer price index	CEIC
Currency Internationalization (INT)	Financial Internationalization (FI)	The overall indicator of financial globalization	KOF Swiss Economic Institute
	Trade Internationalization (TI)	The overall indicator of trade globalization	KOF Swiss Economic Institute

### 3.3 Sampling

This study concentrates on 9 of the top 10 most-used currencies in the global financial market during the period from 2000 to 2020. The selected currencies are the Australian Dollar, Canadian Dollar, Chinese RMB, U.S. Dollar, U. K Pound Sterling, Japanese Yen, Swiss Franc, Sweden Krona, and Hong Kong Dollar. Euro is excluded because some indicators are published at the country level, and relevant data for the European Union during the required period is unavailable. Except for China, all the other issuers of the selected currencies are developed countries. The inclusion of these currencies is justified for several reasons. First, they are all among the top 10 most widely used currencies in the world financial market and are extensively used for cross-border trade, investment, and settlement, providing insights into optimal foreign exchange reserve allocation and risk mitigation. Second, the issuing countries of these currencies are major economies, playing an indispensable role in the operation of the world economy and significantly influencing international financial stability. Third, prior literature usually analyzes the main reserve currencies in the SDR basket, potentially limiting the accuracy of results. This study increases the sample size by including safe-haven currencies (like the Japanese Yen and Swiss Franc), commodity-linked currencies (like the Australian Dollar and Canadian Dollar), emerging market currencies (like the Chinese Renminbi), and traditional international currencies (e.g., U.S. Dollar, British Pound). Expanding the sample provides a more comprehensive understanding of the drivers influencing currency internationalization and better reflects the complexities of global currency dynamics.

### 3.4 Data analysis

The data will be analyzed by using the PLS-SEM method to analyze the complex relationships between the exogenous variables (economic development, money confidence, financial market), the mediator (purchasing power), and the endogenous variable (currency internationalization). The PLS-SEM method normally involves two parts. The first part is to evaluate the formative measurement model (the outer model), which examines the relationships between formative constructs and their respective indicators. Unlike reflective models, the indicators in the formative measurement model are not interchangeable and the relationships between these indicators can be positive, negative, or even not correlated. In this paper, since latent constructs, ED, MC, FM, PP, and INT, are impossible to measure directly, they should be represented by their corresponding indicators.

The formative measurement model assessment consists of examining collinearity and evaluating outer weight and significance to ensure the validity of the constructs. The PLS-SEM algorithm calculates the Variance Inflation Factor (VIF) values for multicollinearity testing while the outer weight and significance are determined by using a one-tail bootstrapping test. To enhance the precision of this study, the bootstrapping process is conducted by using 10,000 subsamples instead of 5,000 subsamples. Indicator collinearity represents the correlations between indicators within constructs, where higher correlations can result in multicollinearity issues. If VIF >  10, it means a high risk of a multicollinearity problem. Some papers select an acceptable threshold of 5 while others encourage to use a stricter criterion with a VIF value of 3.3 or less to better prevent multicollinearity [51]. The weight of an indicator indicates how much contribution it makes to its construct. This implies that indicators with higher outer weights have greater influences on identifying their constructs. The statistical significance of the indicator weights is always determined by T statistics and p values.

The second part is to test the structural model (the inner model), evaluating the relationships between different constructs and testing the hypotheses proposed in Part 2. This includes five steps, which are collinearity (VIF), path coefficient (*β*), coefficient of determination ($R^{2}$), coefficient of effect size ($f^{2}$), and predictive relevance ($Q^{2}$). The criteria for VIF and path coefficient significance in the structural model are the same as those in the formative measurement model. The path coefficient identifies the strength and direction of the direct relationship between latent variables. The sign of the path coefficient indicates the positive or negative relationship between an exogenous variable and an endogenous variable. The coefficient of determination ($R^{2}$) is a measure to examine how much variance in the dependent variable can be explained by other independent variables in the structural model. It is employed to evaluate the predictive accuracy, indicating how well the model fits the observed data. $R^{2}$ values range from 0 to 1, where 0 denotes no predictive accuracy and 1 means total predictive accuracy. A higher $R^{2}$ means a better fit of the structural modal and a higher level of predictive accuracy [17]. Chin proposed three criteria [52], which are 0.67, 0.33, and 0.19 respectively, to determine substantial, moderate, and weak levels of predictive accuracy, while Hair set the criteria at 0.75, 0.5, and 0.25 instead [17]. The coefficient of effect size $f^{2}$ is examining the relative effect of an exogenous variable on an endogenous variable. It is classified into four categories: no impact, small effect, medium effect, and large effect with thresholds of 0.02, 0.15, and 0.35 respectively [53]. Blindfolding is used to determine predictive relevance ($Q^{2}$). $Q^{2}$ reflects the structural model’s power to predict the endogenous variable from the exogenous variables. $Q^{2}$ >  0 indicates that exogenous variables have predictive importance for the endogenous variable. The values 0.02, 0.15, and 0.35 serve as standards for dividing predictive power into weak, moderate, and strong.

Additionally, the mediation effects of purchasing power on the relationships between economic development and currency internationalization and money confidence and currency internationalization are also tested by using a two-tail bootstrapping test with the 10,000 subsamples. When utilizing the bootstrapping technique to examine the mediation effect, it is also needed to consider confidence intervals. The 95% bootstrap confidences must not include the value 0 within the limits of their lower and upper bounds [54]. The formative measurement model, structural model, and mediating effects should all meet the criteria. Table 2 lists the models, assessment, and acceptable values for data analysis.

**Table 2:** The models, assessment, and acceptable values for data analysis.

Model	Assessment	Acceptable Value	
Formative Measurement Model	Collinearity (VIF)	VIF < 10 or VIF < 5 or VIF < 3.3	
	Outer Weight and Significance	P Value ≤ 0.05	
Structural Model	Collinearity (VIF)	VIF < 10;VIF < 5;VIF < 3.3	
	Path Coefficient (β)	P Value ≤ 0.05	
	Coefficient of Determination (${\text{R}}^{2}$)	0.67 $\leq {\text{Q}}^{2}$ - Substantial 0.33 $\leq {\text{Q}}^{2}<$ 0.67 - Moderate 0.19 $\leq {\text{Q}}^{2}<$ 0.33 - Weak ${\text{Q}}^{2}<$ 0.19	0.75 $\leq {\text{Q}}^{2}$ - Substantial 0.50 $\leq {\text{Q}}^{2}<$ 0.75 - Moderate 0.25 $\leq {\text{Q}}^{2}<$ 0.50 - Weak ${\text{Q}}^{2}<$ 0.25
	Coefficient of Effect Size (${\text{f}}^{2}$)	0.35 $\leq {\text{f}}^{2}$ -Large 0.15 $\leq {\text{f}}^{2}<$ 0.35 - Medium 0.02 $\leq {\text{f}}^{2}<$ 0.15 - Small	
	Predictive Relevance (${\text{Q}}^{2}$)	0.35 $\leq {\text{Q}}^{2}$ - Strong 0.15 $\leq {\text{Q}}^{2}<$ 0.35 - Moderate 0.02 $\leq {\text{Q}}^{2}<$ 0.15 -Weak	
Mediation Effect	Significance	P Value ≤ 0.05	
	Confidence Interval	Not include the value 0 within the limits of lower and upper bounds	

### 3.5 Methodology justification

This paper adopts the PLS-SEM method to examine the relationships between latent variables and conduct relevant mediation analysis. This approach is chosen for the following reasons. First, PLS-SEM has fewer limits, particularly regarding data distribution and sample size. It is suitable to handle small sample data and non-normally distributed data, but the outcomes are just as trustworthy as those produced by using covariance-based structural equation modeling (CB-SEM) [17]. Second, the main aim of this paper is consistent with the research purpose of the PLS-SEM method. The PLS-SEM method aims to identify key drivers and extend the existing theory while the CB-SEM method is to test a theory or compare a theory with another theory [18]. Thirdly, using PLS-SEM, it is useful to assess a single indicator in a construct, solve complicated research models, and handle both formative and reflective measurement models. The relationships between indicators with secondary data and constructs are commonly formative measurement models, while CB-SEM can only deal with reflective measurement models. The relationships among the indicators in the formative measurement model can be positive, negative, or even not correlated while indicators in the reflective measurement model are highly correlated [51]. PLS-SEM is flexible with its assumptions, consistent with the research objectives, and adaptable to various research contexts.

## 4. Results and findings

### 4.1 Descriptive statistics

Table 3 shows the means, medians, and standard deviations for all indicators. It presents the basic results of 189 observations from 9 currency issuers between 2000 and 2020. Due to data standardization, all the data are in a specific range from 0 to 1. KAOPEN has the highest mean value of 0.833, indicating a greater average degree of openness in these places. The GR and SMC have extremely low averages of 0.109 and 0.104 respectively, indicating a lower amount of gold reserves, and the stock market is modestly undervalued in these places. Except for GDPS, GDP, and FXR, the variances between means and medians are minimal, smaller than 0.1, implying that the data are evenly distributed around the central point. KAOPEN has the largest standard deviation at 0.308, suggesting notable diversity in openness among the places examined. In contrast, with the lowest standard deviation at 0.129, the numbers for GGR tend to cluster closely around the mean.

**Table 3:** The descriptive statistics for all the indicators in this paper.

Construct	Indicator	Mean	Median	Standard Deviation
Economic Development (ED)	GDP Share (GDPS)	0.180	0.062	0.251
	GDP Growth Rate (GGR)	0.538	0.527	0.129
	FDI (FDI)	0.442	0.395	0.135
	Real GDP (GDP)	0.169	0.064	0.240
Money Confidence (MC)	Gold Reserve (GR)	0.109	0.049	0.156
	Foreign Exchange Reserve (FXR)	0.111	0.011	0.211
Financial Market (FM)	Private Credit to GDP (PC)	0.355	0.339	0.194
	Stock Market Capitalization/GDP (SMC)	0.104	0.056	0.162
	KAOPEN (KAOPEN)	0.833	0.912	0.308
	M2/GDP(M2)	0.190	0.118	0.205
Purchasing Power (PP)	Consumer Price Index (CPI)	0.696	0.704	0.208
	Big Mac Index (BMI)	0.352	0.333	0.222
	Producer Price Index (PPI)	0.744	0.797	0.209
Currency internationalization (INT)	Financial Internationalization (FI)	0.697	0.729	0.273
	Trade Internationalization (TI)	0.642	0.670	0.224

### 4.2 Formative measurement model assessment

**Fig 2:**
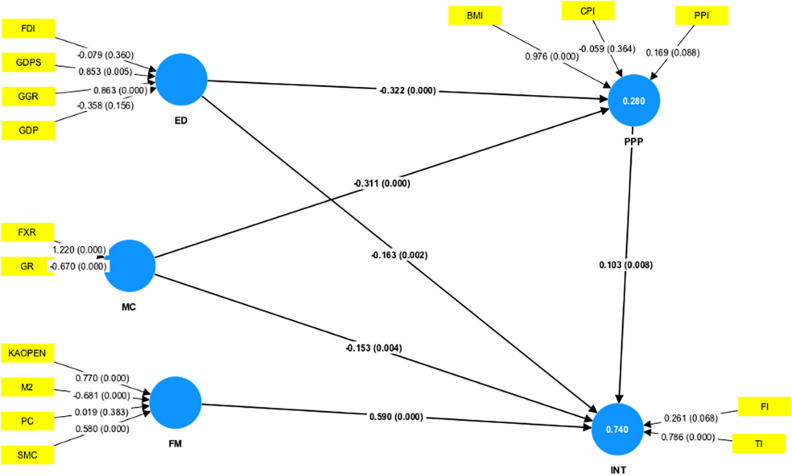
The results of formative measurement model analysis.

Fig 2 shows the results of the formative measurement model analysis and Table 4 depicts the results of the outer weights and significance and VIF values of the formative measurement model. The VIF values for all indicators in different constructs are all smaller than the threshold of 10 and all other indicators meet the stricter VIF criterion of being below 5, except for GDPS and GDP, which have VIF values of 7.209 and 8.455, respectively. This phenomenon means a lower risk of multicollinearity problems in each construct, which suggests that the indicators are not excessively correlated with one another, the model is quite stable, and the outcomes are trustworthy.

**Table 4:** The outer weights, significance, and VIF values of the formative measurement model.

Indicator	Original Sample	Sample Mean	Standard Deviation	^T^ Statistic	P Value	VIF	Supported
GDPS - > ED	$0.853^{\text{***}}$	0.879	0.334	2.558	0.005	7.209	YES
GGR - > ED	$0.863^{\text{***}}$	0.834	0.081	10.638	0.000	1.062	YES
FDI - > ED	$-0.079^{NS}$	−0.086	0.220	0.357	**0.360**	2.258	NO
GDP - > ED	$-0.358^{NS}$	−0.380	0.354	1.012	**0.156**	8.455	NO
GR - > MC	$-0.670^{\text{***}}$	−0.691	0.148	4.527	0.000	1.489	YES
FXR - > MC	$1.220^{\text{***}}$	1.220	0.128	9.550	0.000	1.489	YES
PC - > FM	$0.019^{NS}$	0.022	0.065	0.299	**0.383**	1.201	NO
SMC - > FM	$0.580^{\text{***}}$	0.588	0.132	4.395	0.000	4.495	YES
KAOPEN - > FM	$0.770^{\text{***}}$	0.762	0.051	15.012	0.000	1.254	YES
M2 - > FM	$-0.681^{\text{***}}$	−0.679	0.123	5.548	0.000	4.069	YES
CPI - > PP	$-0.059^{NS}$	−0.066	0.170	0.348	**0.364**	1.373	NO
BMI - > PP	$0.976^{\text{***}}$	0.959	0.062	15.797	0.000	1.139	YES
PPI - > PP	$0.169^{\text{*}}$	0.170	0.125	1.351	0.088	1.256	WS
FI - > INT	$0.261^{\text{*}}$	0.283	0.175	1.492	0.068	2.431	WS
TI - > INT	$0.786^{\text{***}}$	0.759	0.158	4.963	0.000	2.431	YES

**Note(s):** NS represents p-value > 0.1; *  represents p-value ≤  0.1; ** represents p-value ≤  0.05; *** represents p-value ≤  0.01 (one-tailed).

NS: Not significant; YES: Supported with p-value ≤  0.05 or 0.01; WS: Weak supported with p-value >  0.05 or < 0.1; NO: Not supported.

Within the ED construct, GDPS and GGR are statistically significant with p-values of 0.005 and 0 and T-statistics of 2.558 and 10.638 respectively at a 1% significance level. Both GDPS and GGR have positive weights (0.853 and 0.863), indicating that an increase in GDPS and GGR correlates with an increase in economic development. Conversely, FDI and GDP do not satisfy statistical significance, showing p-values of 0.360 and 0.156 respectively. Given p values of 0, the two indicators in the MC construct, GR and FXR, are of statistical significance. The positive weight for FXR shows that the growth in foreign currency reserves is correlated with the growth in money confidence, whereas the negative weight for GR indicates greater gold reserve leads to lower money confidence. For the FM construct, three indicators, SMC, KAOPEN, and M2, are statistically significant at a 1% significance level while the t-statistics range from 4.395 to 15.012. M2 has a negative weight, indicating that a decrease in M2 corresponds to an increase in the financial market, while SMC and KAOPEN have positive weights, showing opposite relationships. In the mediation construct PP, BMI, and PPI are significant at 1% and 10% respectively and both show positive relationships with purchasing power. In the endogenous variable INT, the indicator FI is only significant at 10% and TI has a significant relationship with INT at 1%. Both FI and TI have positive relationships with the construct INT.

### 4.3 Structural model assessment

**Fig 3:**
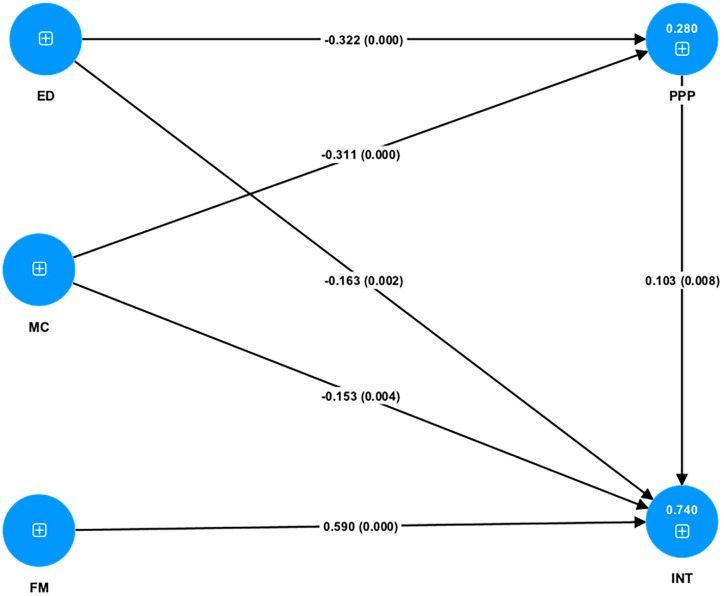
The results of structural model analysis.

Fig 3 shows the results of structural model analysis and Table 5 shows the results of the hypotheses, path coefficients, VIF values, effect size ($f^{2}$), coefficient of determination ($R^{2}$), predictive relevance ($Q^{2}$) and mediation effect. The first step is to assess the collinearity by comparing VIFs. The VIF values of constructs are all below 5 and even below 3.3, implying a very low potential for multicollinearity issues between these constructs [50]. The second step is to analyze path coefficients and significance. When the mediator variable PP is included, all hypotheses from H1 to H6 are statistically significant at the significance level of 1%. The path coefficients show that ED and MC have negative relationships with INT, while FM has a positive relationship with INT. PP also has a significant relationship with INT, with a p-value of 0.008, indicating that the higher purchasing power of a currency supports its currency internationalization process. What’s more, ED and MC negatively affect PP, while PP positively influences INT, suggesting that while economic development and market confidence may lower currency purchasing power, greater purchasing power contributes positively to international currency expansion. The third step is to analyze the coefficient of determination ($R^{2}$). The $R^{2}$ value for the PP construct is 0.28, signifying that about 28% of the variance in PP could be explained by ED and MC. This model demonstrates a weak effect on predictive accuracy [51], suggesting that other unexamined variables may contribute to PP. In contrast, the $R^{2}$ value INT is significantly higher at 0.74, meaning 74% of the variance in currency internationalization can be explained by this model. This demonstrates a strong predictive accuracy for international currency, meaning that the model successfully captures the key factors driving internationalization. The fourth step is to evaluate effect size $f^{2}$. The results show that economic development and money confidence have small effects on currency internationalization, with effect sizes of 0.072 and 0.039, suggesting their influence on internationalization is limited. The financial market has a large effect size with the $f^{2}$ value of 0.535, surpassing the threshold of 0.35 dramatically, which means a sound financial market plays a significant role in driving currency internationalization. Purchasing power has a small effect on INT with an effect size of 0.029, indicating a minimal direct influence. In terms of the relationships between ED on PP and MC on PP, both have small effect sizes with 0.121 and 0.113 respectively, implying that these two factors have a relatively weak impact on purchasing power. The main finding is the significant role of the financial market in promoting currency internationalization, while the effects of economic development, money confidence, and purchasing power are comparatively smaller. The fifth step is to examine predictive relevance ($Q^{2}$). The $Q^{2}$ value for PP is 0.116, bigger than 0.02 and smaller than 0.15, suggesting that if purchasing power is regarded as the dependent variable, the two independent variables, economic development and money confidence have weak predictive relevance. This implies although these two exogenous variables can explain some variance in purchasing power, their predictive power is relatively limited. In contrast, the $Q^{2}$ value for INT is 0.639, greater than 0.35, indicating a stronger power of predictive relevance. This means the whole model, including purchasing power as a mediator, provides a good fit for predicting the currency internationalization process.

**Table 5:** The results of path coefficients, VIF values, effect size $f^{2}$ coefficient of determination $R^{2}$ predictive relevance $Q^{2}$ and mediation effect.

Direct Effect	Path Coefficient	Sample Mean	Standard Deviation	T Statistic	P Value	VIF	$f^{2}$	Effect Size	Supported
H1:ED- > INT	$-0.163^{\text{***}}$	−0.171	0.056	2.930	0.002	1.428	0.072	Small	Yes
H2:MC- > INT	$-0.153^{\text{***}}$	−0.154	0.058	2.652	0.004	2.273	0.039	Small	Yes
H3:FM- > INT	$0.590^{\text{***}}$	0.585	0.069	8.589	0.000	2.506	0.535	Large	Yes
H4:PP- > INT	$0.103^{\text{***}}$	0.102	0.042	2.426	0.008	1.417	0.029	Small	Yes
H5:ED- > PP	$-0.322^{\text{***}}$	−0.332	0.064	5.039	0.000	1.188	0.121	Small	Yes
H6:MC- > PP	$-0.311^{\text{***}}$	−0.322	0.081	3.822	0.000	1.188	0.113	Small	Yes
$R^{2}$ Values: PP=0.280; INT=0.740 $R^{2}$ Adjusted Values: PP=0.272; INT=0.735 $Q^{2}$ Values: PP = 0.116 weak predictive power; INT = 0.639 strong predictive power									
**Indirect Effect**	**Path Coefficient**	**Sample Mean**	**Standard Error**	**Confidence Interval (LL)**		**Confidence Interval (UL)**	T **Statistic**	**P Value**	**Supported**
H7:ED- > PP- > INT	$-0.033^{\text{**}}$	−0.034	0.016	−0.067		−0.005	2.100	0.036	YES
H8:MC- > PP- > INT	$-0.032^{\text{**}}$	−0.032	0.016	−0.066		−0.006	2.029	0.042	YES

Note(s) ED: Economic Development; MC: Money Confidence; FM: Financial Market; PP: Purchasing Power; INT: Currency internationalization.

NS represents p-value > 0.1; *  represents p-value ≤  0.1; ** represents p-value ≤  0.05; *** represents p-value ≤  0.01 (two-tailed).

NS: Not significant; YES: Supported with p-value ≤  0.05 or 0.01; WS: Weak supported with p-value >  0.05 or < 0.1; NO: Not supported.

### 4.4 Mediation effect assessment

As shown in Table 5, the results reveal that purchasing power plays a significant mediating role in the relationships between economic development and currency internationalization and money confidence and currency internationalization. PP positively influences INT (β =  0.103, p =  0.008), while both ED and MC have direct negative impacts on INT (β =  −0.163, p =  0.002; β =  −0.153, p =  0.004 respectively) and on PP (β =  −0.322, p =  0.000; β =  −0.311, p =  0.000). The mediation effects of PP in both relationships are statistically significant at the significance level of 5% (ED- > PP- > INT: β =  −0.033, p =  0.036; MC- > PP- > INT: β =  −0.032, p =  0.042). This indicates that the mediation effect of PP on ED and INT and on MC and INT are both partial mediations rather than full mediation. This means that a portion of the influence of economic development and money confidence on currency internationalization is transmitted indirectly by purchasing power. The negative products of the path coefficients for the relationships ED- > PP, PP- > INT, and ED- > INT (−0.322 *  0.103 *  −0.163) and for MC- > PP, PP- > INT, and MC- > INT (−0.311 *  0.103 *  −0.153) indicate the complementary mediation effects in both cases. Therefore, hypotheses H7 and H8 are both supported and purchasing power mediates the relationships between ED- > INT and MC- > INT. This finding suggests that economic development and money confidence may negatively influence purchasing power, thereby weakening the currency’s ability to act as an international currency. It highlights the complex dynamics between economic performance factors and currency internationalization.

## 5. Discussion

To reduce currency risks and protect national interests, countries, especially some emerging nations, are pursuing greater international currency diversification and reducing their reliance on a single dominating currency in the face of dedollarization and deglobalization sentiments. This paper aims to explore international currency operation mechanisms and find the key drivers of currency internationalization. By analyzing data from nine international currency issuers, this study investigated the direct effects of economic development, money confidence, and the financial market on currency internationalization. The indirect effects of mediator purchasing power on economic development and currency internationalization and money confidence and currency internationalization were also examined. However, upon reviewing the relevant literature, it is found that the study conducted by Cui et al. is the most closely related to this paper. A detailed evaluation of these two papers presents some major differences. There are two main differences as below.

First, Cui et al. [32] focused more on the reserve currencies in the SDR basket and used multiple regression models to examine results while this paper analyzes 9 of the 10 most used currencies by using the PLS-SEM method. In this paper, three corresponding hypotheses H1, H2, and H3 are all statistically significant. Except for the aspect of money confidence, not addressed in Cui et al.’s study, this study similarly identifies significant relationships among economic development, financial markets, and currency internationalization, aligning with their findings. However, Cui et al. [32] introduced a new variable competitiveness as a key driver of international currency, which is not included in this study and gives inspiration for future study. The financial market is positively related to currency internationalization in both studies. The developed financial market offers the depth and liquidity necessary for handling large transactions, which is a crucial aspect of international currencies. A developed financial market tends to be open, transparent, and integrated with the global market, facilitating cross-border capital flows and the use of domestic currencies in international transactions. The development of the financial market contributes to the liberalization of capital accounts enabling the free movement of financial assets across borders. However, the findings that economic development and money confidence have negative relationships with currency internationalization are different from Cui et al [32]. It is because the currencies included are all from advanced economies, characterized by stable economic conditions and consistent growth. However, the international usage of their currencies has reached saturation without great changes, leading to a situation where the currency internationalization process cannot match the economic development of these countries. Meanwhile, developed economies tend to hold various currencies for their settlements, trade, and transactions to mitigate exchange rate risks because they prefer to use a currency diversification strategy rather than depend on their currencies as an effective way of risk management. Money confidence also has a negative relationship with currency internationalization, different from previous studies [3,32,40]. This is mostly because of the indicators that money confidence included. Typically, foreign exchange reserves and gold reserves are utilized to protect a country from currency risks. If a country prioritizes its gold and foreign exchange reserves, it signals a tendency towards risk aversion, emphasizing the preservation of economic stability and currency value. This is in opposition to the flexibility and openness needed for currency internationalization. Another reason is that the country prefers to use these assets to uphold equilibrium in international payments rather than depend heavily on domestic currency transactions.

Second, Cui et al. [32] only evaluated the direct relationships between key drivers and currency internationalization while this study explores whether mediation effects of purchasing power exist on the relationships between the first two drivers and currency internationalization, which is not included in prior studies. Five corresponding hypotheses from H4 to H8 are all supported. Purchasing power has a positive relationship with currency internationalization, which is in accordance with [11]. If a currency has more purchasing power, it means this money has a higher value and it can be used to purchase goods and services both domestically and internationally, which makes it attractive to international investors [10] Economic growth driven by innovation causes increased purchasing power through the creation and redistribution of financial resources [48] while this study finds that economic development has a negative relationship with purchasing power is different from previous papers [12]. Money confidence also has a negative relationship with purchasing power in this study, which is consistent with previous papers related to precious metals [6,41]. The reasons for these results are proposed as follows. Economic development is usually accompanied by increased demand and rising prices, leading to inflation. Inflation causes a country’s currency to depreciate, reducing its purchasing power. Economic development will increase the domestic demand for foreign products and services. Among the countries involved in the study, except for Australia, Switzerland, and China, most countries had witnessed trade deficits. Their imports exceeded their exports, and they need to pay more foreign currency to afford imported goods and services. This leads to an increase in demand for foreign currency and a decrease in demand for domestic currency, resulting in a devaluation of the domestic currency and a decrease in its purchasing power. In terms of reserve assets related to money confidence, developed countries tend to hold smaller foreign exchange reserves and gold reserves than developing countries [55]. The decrease in gold reserves indicates less uncertainty or reduced inflationary pressures in the economy while higher foreign exchange reserves means that the country has enough reserves to maintain the stability of its currency in the face of external shocks. For example, Japan from 2003 to 2004 and Switzerland from 2009 to 2010 both intervened aggressively to prevent their currencies from appreciating too much during that time [56]. Since most of the economies in the study had persistent trade imbalances, with their imports exceeding their exports for a long time, a currency depreciation policy is a regular and necessary measure to encourage their exports.

## 6. Conclusion and policy recommendation

### 6.1 Conclusion

Currency internationalization has attracted great interest due to the tendency of dedollarization and deglobalization. This study tries to examine the key drivers of currency internationalization and the complex mechanisms behind international currency by using the PLS-SEM method. Based on the data for 9 of the 10 most used currency-issuing countries between 2000 to 2020, this study reveals that economic development, money confidence, and financial market have significant direct relationships with currency internationalization. The financial market has a large effect size among the three exogenous variables, while economic development and money confidence have a small effect size. The findings of the study reveal that economic development and money confidence in advanced countries have a negative and significant effect on purchasing power and currency internationalization. However, the financial market has a positive and significant effect on the international currency, suggesting the development of the financial market greatly promotes the currency internationalization process. What’s more, purchasing power has a positive relationship with currency internationalization and it acts as a mediator in relationships between economic development and currency internationalization and money confidence and currency internationalization. Both mediation effects are complementary mediation effects, implying that purchasing power partially explains how economic development and money confidence influence currency internationalization, but there are other direct or indirect pathways influencing currency internationalization besides PP.

### 6.2 Research contribution

This study enhances the literature on currency internationalization by analyzing data from nine international currency issuers. Previous papers focused more on the reserve currencies in the SDR basket, while this study expands the scope by considering additional currencies, including those with the potential to become reserve currencies. It establishes a model of currency internationalization based on CS theory and PPP theory and explores the connections between economic development, money confidence, financial market, and currency internationalization. It also examines how purchasing power mediates the relationships between economic development and currency internationalization and money confidence and currency internationalization.

Furthermore, this study contributes to the literature on macroeconomic performance by highlighting the significance of economic development, money confidence, and the financial market in the process of currency internationalization. It suggests that for advanced economies, the increase in economic development and money confidence may not promote the process of currency internationalization and can even impair the process due to economic regulation. This insight provides valuable guidance for some emerging economies, such as China and India, to promote the process of currency internationalization.

The method effectively analyzes the complex relationships between exogenous variables (economic development, money confidence, financial markets), the mediator (purchasing power), and endogenous variable (currency internationalization). The complex interactions of these latent variables, as well as how they affect the currency internationalization process have never been studied by using this method before.

### 6.3 Policy recommendations

According to the main findings of the study, some practical policies are proposed for the promotion of currency internationalization. The results indicate that an open, deep, and well-developed financial market contributes to the process of currency internationalization. Since SMC, KAOPEN, and M2, are statistically significant in financial market construct. Countries should give priority to enhancing market depth by developing financial infrastructure, supporting the growth of local stock exchanges, and improving regulatory frameworks. Meanwhile, encouraging financial market openness will increase transparency, accessibility, and integration with the global financial market. Countries, like China, that have not yet fully opened up capital accounts need to reduce capital controls, attract foreign investments, and enhance the free flow of capital across their borders. Furthermore, central banks should notice the significance of monetary aggregates and sustain a balanced stable monetary policy. In addition, economic development and money confidence negatively influence currency internationalization for the developed economies involved. This suggests economic development and increased reserve assets can sometimes lead to currency depreciation. Governments had better maintain stable currency value by controlling inflation through monetary and fiscal policies, diversifying reserve asset management to avoid over-reliance on foreign exchange and gold reserves and promote a flexible exchange rate system to manage the outer value of currency.

### 6.4 Limitations and suggestions for future studies

Three limitations are covered in this study, which provides some enlightenment for future study. First, as one of the most essential international currencies, the euro is not covered in this study due to unavailable data. Many indicators, like gold reserve and KAOPEN, do not include the data of the whole European Union. Second, the data for all indicators are hard data and no soft data are included. The concept of money confidence is always related to people’s trust and feelings, which is better captured by soft data. Furthermore, owing to research capability, the sample size is small only consisting of 9 currencies for 21 years. Long-term observations will be more convincing. Third, by using the PLS-SEM method, more constructs can be considered and added to the conceptual model. The three independent variables in this study are all economic factors, and there are many other factors affecting currency internationalization, including politics, culture, and military, which also should be taken into the conceptual framework.

To summarize, in terms of currency internationalization, economic development, money confidence, financial market, and purchasing power are all essential factors. This paper addressed the gap in empirical research by examining the key drivers, the mediator purchasing power, and currency internationalization in a conceptual framework and extended the application scope of the PLS-SEM method, especially in dealing with secondary data in international finance and economics. Therefore, this study made great contributions to economic performance and international currency.

## Supporting information

S1 Data Data availability. (XLSX)
